# Enrichment of anammox bacteria in up-flow bioreactors enhanced with plastic and rock media: Long-term performance monitoring with fluorescence and specific conductivity

**DOI:** 10.1016/j.biteb.2025.102081

**Published:** 2025-03-08

**Authors:** Polina Popova, Lilith Astete Vasquez, Shiloh Bolden, Natalie Mladenov

**Affiliations:** a Department of Civil, Construction, and Environmental Engineering, San Diego State University, 5500 Campanile Dr., San Diego, CA 92182, USA; b Now at: Department of Chemical and Environmental Engineering, Yale University, New Haven, CT 06520, USA

**Keywords:** Anammox, Up-flow bioreactors, Biocarriers, Fluorescence, Specific conductivity

## Abstract

Anaerobic ammonium oxidation (anammox) is a low energy, biological process for removal of nitrogen from wastewater. Here we tracked anammox enrichment and recovery after environmental disruption using two rapid monitoring techniques, fluorescence and specific conductivity, and compared the ability of two readily available media types, plastic (PMR) and lava rock (RMR), to serve as biocarriers for anammox in low-maintenance, up-flow bioreactors. We found that both PMR and RMR became enriched with primarily *Candidatus brocadia*-dominated microbial consortia. Optimal (> 90 %) ammonium-N and nitrite-N removal was accomplished more rapidly in PMR (after 147 d) than in RMR (after 171 d). However, after long-term operation, RMR was more resilient to disruption than PMR. In both reactors, the increase in effluent/influent ratio of the Freshness Index, and the reduction in specific conductivity from influent to effluent were significantly correlated with ammonium uptake during the start-up phase but not in the stable phase.

## 1. Introduction

The anaerobic ammonium oxidation (anammox) process has been increasingly used for sidestream treatment of centrate and other ammonium (${\text{NH}}_{4}^{+}$)-rich reject waters at wastewater treatment facilities (Kosari et al., 2013; Kosgey et al., 2020) as an alternative to coupled nitrification/denitrification (N/D). Foundational works of van de Graaf et al. (1995) and Strous et al. (1998) describe methods for anammox enrichment in sequencing batch reactors (SBRs), and subsequent studies have further addressed optimal and inhibiting conditions for anammox growth (Egli et al., 2001; Dapena-Mora et al., 2007; Jin et al., 2012). Even when optimal conditions are maintained rigorously, anammox duplication time is slow, at 7–20 days (Adams et al., 2020). Various methods for successfully expediting anammox enrichment have been tested, including increasing biomass retention with biomass carrier amendments, such as volcanic rock, plastic media, ceramics, and other materials (Ganesan and Vadivelu, 2019; Ma et al., 2024; Ren et al., 2014; Tuyen et al., 2018; Yang et al., 2021; Zhang et al., 2010). Volcanic or lava rock and plastic carriers, which are inexpensive and readily available for anammox enrichment studies, have received contrasting reports regarding their ability to support anammox biomass (Fofana et al., 2022; Gao et al., 2022). Side-by-side comparisons of identical reactors treating the same wastewater or synthetic feed solution, differing only in their carrier material, are needed to better assess carrier effectiveness.

Enrichment reactors include sequencing batch reactors, expanded granular sludge bed (EGSB) reactors, membrane bioreactors (MBRs), and continuous stirred tank reactors (CSTRs) (Li et al., 2014; Lin et al., 2024; Strous et al., 1998; Zhang et al., 2022), which promote anammox formation in granules, and moving bed biofilm reactors (MBBRs), packed bed reactors (PBRs), and anaerobic baffled reactors (ABRs) (Duarte et al., 2021; Gustavsson et al., 2020; Rivera et al., 2022), which promote biofilms that house a consortium of anammox and other microorganisms that support the anammox process (Zhao et al., 2018). There has been less attention paid to up-flow reactors containing biomass carriers for biofilm growth, which have lower programming requirements and may, therefore, be more easily maintained. Research into anammox enrichment using readily available biofilm carriers and low-maintenance methodologies is warranted, especially for decentralized applications. Also, the start-up of anammox is a fragile process that calls for real-time monitoring to track growth during start-up and enable swift responses in case of anammox inhibition.

Monitoring of effluent water quality, in particular the effluent dissolved organic matter (EfDOM), provides insight into the metabolic processes within an anammox reactor because EfDOM contains diverse compounds associated with biomass growth and decomposition and has been widely used as a proxy for specific microbial presence and activity (Kuo and Parkin, 1996). Several methods have been used to characterize EfDOM, such as total organic carbon (TOC) analysis to measure the overall quantity of organic matter present, DuBois and Lowry methods to determine polysaccharide, protein, and humic substance levels, and DAX (or XAD) resin column chromatography to separate hydrophobic and hydrophilic components for further analyses, but these are time consuming and laborious laboratory techniques (Yu et al., 2020). Fluorescence spectroscopy has been used to track anammox enrichment (Hou et al., 2017; Ruscalleda et al., 2014) and may offer a more practical approach due to comparatively lower cost, time, and labor requirements than other EfDOM characterization methods. Similarly, specific conductivity, a measure of dissolved free ions in solution, like ammonium and nitrite, was successfully used to track anammox enrichment in up-flow anaerobic sludge blanket reactors (Li et al., 2012; Yu and Jin, 2012). However, studies have been relatively short, limited mostly to start-up periods, and the use of specific conductivity as a metric during steady-state operation and during inhibition periods warrants further investigation.

Therefore, the primary aims of this study were to 1) evaluate rapid monitoring techniques, such as fluorescence and specific conductivity, for tracking anammox enrichment and the recovery of anammox after environmental disruption and 2) compare the ability of two media types, plastic and lava rock, to serve as biocarriers for anammox in low-maintenance, up-flow bioreactors. Fluorescence and specific conductivity were tracked and evaluated as performance indicators during start-up phase, under steady state, and during inhibition events for ~500 days of operation. In addition, we measured dissolved organic carbon, total dissolved nitrogen, ${\text{NH}}_{4}^{+}-\text{N},\;{\text{NO}}_{2}^{-}-\text{N}$, and ${\text{NO}}_{3}^{-}-\text{N}$ concentrations in the feed and effluent of the reactors, tracked stoichiometric relationships, and conducted high throughput DNA sequencing to confirm the presence of anammox bacteria.

## 2. Methods

### 2.1. Experimental setup

Each of the up-flow media reactors was constructed using an Aqua-Maxx T-X Secondary Media Chamber for Calcium Reactors, Model FR-SE (cost of US$80 in 2024; diameter: 8.1 cm; height: 37.1 cm). Cylindrical polyester plastic hair rollers (Conair brand) were stacked vertically in a concentric configuration inside the plastic media reactor (PMR), resulting in ~1.2 L of working volume. Lava rock was used to fill the rock media reactor (RMR), resulting in a working volume of ~0.83 L. The reactors were wrapped with heat mats to raise their internal temperature to ~27 °C.

Prior to the current study, PMR was used in a 3-month long period of anammox troubleshooting, during which optimal feed and operational procedures for anammox growth were established. Biomass containing anammox bacteria from Chamber 1 of a lab-scale anammox-enrichment ABR (Rivera et al., 2022) containing stabilized sludge from San Elijo Water Reclamation Facility was used in initial inoculation of PMR. Two months later, effluent from an anammox enrichment reactor from Los Angeles County Sanitation District was also fed into the PMR to stimulate anammox growth. By the end of the trouble-shooting period, ${\text{NH}}_{4}^{+}$ and ${\text{NO}}_{2}^{-}$ removal in PMR was <6 %. The RMR had no troubleshooting phase and was inoculated using only stabilized sludge from San Elijo Water Reclamation Facility.

PMR and RMR were fed simultaneously in parallel using the same synthetic feed for 536 and 498 d, respectively. The feed solution recipe was adapted from Strous et al. (1998) with the Ca/P ratio lowered to 0.5 (g/g) (Supplemental Table S1) to reduce calcium phosphate precipitation as in Trigo et al. (2006). Tap water used in preparation of the synthetic feed was purged with nitrogen gas (N_2_) to achieve dissolved oxygen concentrations of <0.50 mg/L, and hydrochloric acid (37 %) was used to lower feed solution pH to ~7.5. The synthetic feed was continuously stirred and maintained at ~38 °C using a heated stir plate. The reactor feeding schedule was intentionally designed with intermittent pumping periods (by peristaltic pump) lasting 30 min and reaction periods (no pumping) lasting 2 h that lengthened the time between resupply of synthetic feed. The volumetric flow rate of synthetic feed in PMR and RMR was 0.59 L/h and 0.60 L/h, respectively. This resulted in 7 cycles per day with an 8.5 h rest period to simulate the overnight reduction in wastewater flow rates in decentralized wastewater treatment systems treating domestic wastewater (Mladenov et al., 2017). Consequently, the resupply of feed and maintenance and monitoring were performed only three times per week. This schedule could be even less frequent if a larger feed tank were employed.

On day 473 for PMR, biomass from both PMR and RMR were removed to conduct a specific anammox activity (SAA) test, as described in Rivera et al. (2022). Ammonium, nitrate, and nitrite were analyzed after 24 h, 48 h, and 72 h of incubation to evaluate the presence and N-removal performance of anammox bacteria.

### 2.2. Water quality analyses

Influent and effluent pH, specific conductivity, dissolved oxygen, and temperature were measured three times per week. The pH and specific conductivity were measured using a Fisherbrand Accumet AP85 meter. Dissolved oxygen and temperature were measured using a YSI ProODO Optical dissolved oxygen meter. During the first year of the study, nutrient concentrations were measured weekly. From days 361–536 in PMR and day 324–498 in RMR, performance monitoring was less frequent (biweekly or monthly) due to staffing challenges. Ammonium-N concentrations were determined using a HACH DR3900 spectrophotometer and Hanna Ammonia Checker colorimeter. Ion chromatography with DIONEX Ion Chromatography System Model ICS-2000 and IonPac AG23 (4 mm) column was used for determining nitrate-N and nitrite-N concentrations. Dissolved organic carbon and total dissolved nitrogen were quantified weekly with a Shimadzu TOC-L Total Organic Carbon and Total Nitrogen Analyzer using a high temperature oxidation method.

### 2.3. Fluorescence analyses

Three-dimensional excitation-emission matrix (EEM) spectra were acquired for influent and effluent samples each week with a Horiba Scientific Aqualog Fluorometer using a 1 cm pathlength quartz cuvette. Spectra were acquired at excitation (Ex) wavelengths of 240 nm–450 nm with an increment of 3 nm, emission (Em) wavelengths of 240 nm–800 nm with an increment of 4.66 nm, integration time of 1 s, and high gain. The relationship between nutrient (${\text{NH}}_{4}^{+}$ and ${\text{NO}}_{2}^{-}$) removal and intensities of five commonly reported fluorescent peaks (Coble, 1996), Peak A (Ex/Em = 260 nm/380–460 nm) and Peak C (Ex/Em = 350 nm/420–480 nm), which are associated with humic substances, Peak T (Ex/Em = 275 nm/340 nm), which reflects the presence of tryptophan-like substances, Peak B (Ex/Em = 275 nm/310 nm), which represents tyrosine-like substances, and Peak M (Ex/Em = 312 nm/380–420 nm), which represents marine humic-like substances, were analyzed for surrogacy to anammox growth. Relationships between nutrient removal and freshness index (Em intensity at 380 nm/Em intensity maximum at Em 420–435 nm at Ex 310 nm), indicating the presence of recently produced extracellular dissolved organic matter, and humification index (HIX; area under the Em spectra 435–480 nm/peak area 300–345 nm + 435–480 nm, at Ex 254 nm), indicating the degree of organic matter decomposition and maturity, were also analyzed. Peak F420 (Ex/Em = 420/460–490 nm) was also studied, as several previous studies (Hou et al., 2017; Lu et al., 2017; Ruscalleda et al., 2014) have tracked Peak F420 in relation to anammox growth. MATLAB software version R2023a was used to process raw data as described in Rivera et al. (2022). Correlation analysis between nutrient removal and changes in fluorescent characteristics was conducted using Statistical Product and Service Solutions (SPSS) software.

### 2.4. Microbial community composition

Microbial communities were assessed for each reactor on the same date, corresponding to 202 d of operation for PMR and 164 d for the RMR, to confirm presence of anammox bacteria and evaluate their abundance in concert with mutualistic species. We aimed to collect biomass samples during the stable phase of operation, and this particular sampling date during the stable phase was selected due to student worker availability. After collecting biomass from the top and middle sections of each reactor using sterile pipettes, samples were allowed to settle by gravity. Total DNA was immediately extracted from the settled biomass using DNEasy Power Soil Kit (Qiagen, USA). The isolated DNA was frozen at 4 °C prior to sequencing. Sequencing was conducted by MR DNA Lab (www.mrdnalab.com, Shallowater, TX, USA). Amplification of the 16S rRNA gene V4 variable region was performed using the HotStarTaq Plus Master Mix Kit (Qiagen, USA), followed by preparation of an Illumina DNA library. Sequencing was carried out on a MiSeq platform in accordance with manufacturer protocols. The resulting filtered and denoised zero-radius operational taxonomic units (zOTUs) were classified using BLASTn against a curated NCBI database (www.ncbi.nlm.nih.gov) and species identification was performed by matching sequences sharing ≥97 % similarity.

## 3. Results and discussions

### 3.1. Nitrogen removal performance

**Fig. 1.**
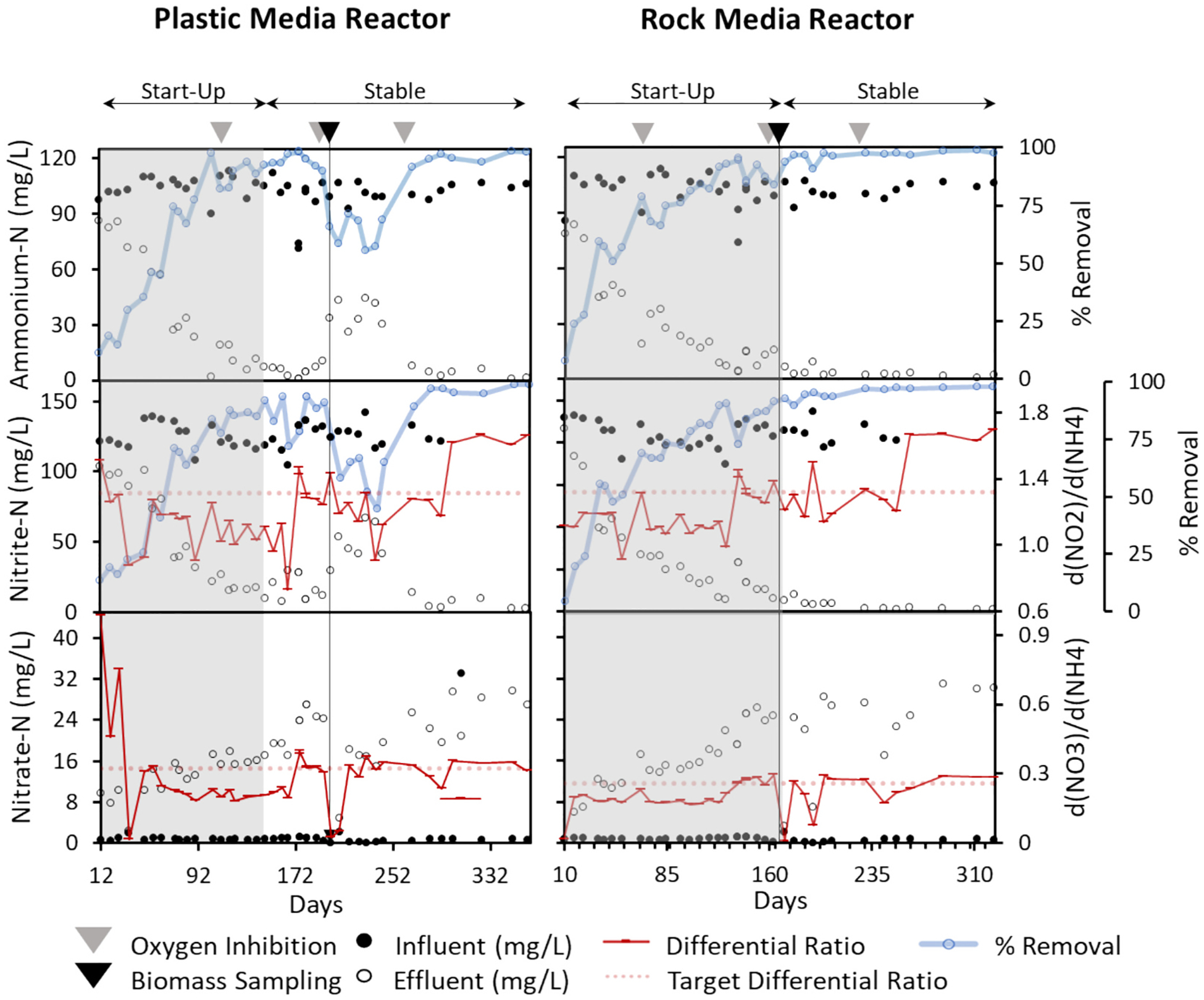
Nitrogen removal performance in plastic and rock media reactors. PMR operation began 38 days before RMR. Therefore, day 10 of RMR and day 48 of PMR represent the same sampling date. Nutrient analysis began on day 12 for PMR and day 10 for RMR.

Over the operating periods of this study (536 d for PMR and 498 d for RMR), ammonium-N and nitrite-N influent concentrations were maintained at ~102 mg/L (7.28 mmol/L) and ~ 130 mg/L (9.28 mmol/L), respectively. Despite several disruptive events during anammox enrichment, each reactor demonstrated improvement in ${\text{NH}}_{4}^{+}-\text{N}$ and ${\text{NO}}_{2}^{-}-\text{N}$ removal over time (Fig. 1). The start-up phase was shorter for PMR (lasting 147 d) compared RMR (lasting 171 d), after which a stable phase with consistent >90 % ${\text{NH}}_{4}^{+}-\text{N}$ removal was successfully maintained. By day 88, both reactors displayed disperse pink-colored microcolonies, and by day 170, both had developed a distinct pink coloration, indicative of anammox biofilms (Supplemental Figs. S1 and S2). We note that more rapid flowrates have been successfully employed in other studies (Li et al., 2012; Qin et al., 2017), which would reduce the start-up times. By the end of the start-up phase, the stoichiometric ratios between removed and generated nutrients in the reactors were 1.11 (PMR) and 1.21 (RMR) for ${\text{NO}}_{2}^{-}/{\text{NH}}_{4}^{+}$ (uptake/uptake) and 0.17 (PMR) and 0.27 (RMR) for ${\text{NO}}_{3}^{-}/{\text{NH}}_{4}^{+}$ (generation/uptake), matching ratios previously reported for anammox metabolism (Strous et al., 1999; Zhang et al., 2018) and supporting the visual evidence of anammox growth within both reactors. These findings suggest that it is possible to enrich anammox using a simple system design and under low maintenance conditions.

Several oxygen inhibition events, where air was accidentally pumped into the up-flow reactors, occurred during both start-up and stable phases that allowed us to test anammox recovery in both reactors. In other reactor types (e.g., Li et al., 2016; Liu et al., 2017; Rivera et al., 2022), exposure to oxygen is not problematic because of the oxygen concentration gradients within biofilms that support a consortium of microbes, including anammox. However, in our sealed up-flow reactor, oxygen addition did pose a disruption, but only during the start-up phase. During start-up (day 105 (PMR) and day 67 (RMR)), oxygen inhibition lasting 15–30 min had a notable negative effect on reactors’ performance, decreasing the ammonium removal from 98 % to 83 % in PMR and from 79 % to 68 % in RMR (Fig. 1). Conversely, during the stable operation phase (day 266 (PMR) and day 228 (RMR)), nutrient removal rates in both reactors were unaffected by 90 min of oxygen inhibition (Fig. 1). These findings suggest that, once enriched, anammox systems can likely withstand oxygen exposure without triggering the system’s collapse. However, disruptions caused by more impactful physical disturbance of the biofilm during biomass sampling for DNA extraction did produce a notable negative effect on system performance in PMR, but not RMR, even during the stable operation phase. The action of dislodging biomass from various areas of the reactor would have been equally disruptive to the microbial communities in both reactors. Therefore, we attribute this reduction in performance (ammonium-N removal reduced to <72 % for 60 days in PMR) to a less resilient biomass on the plastic media, as has been suggested in other studies (Yang et al., 2021).

**Fig. 2.**
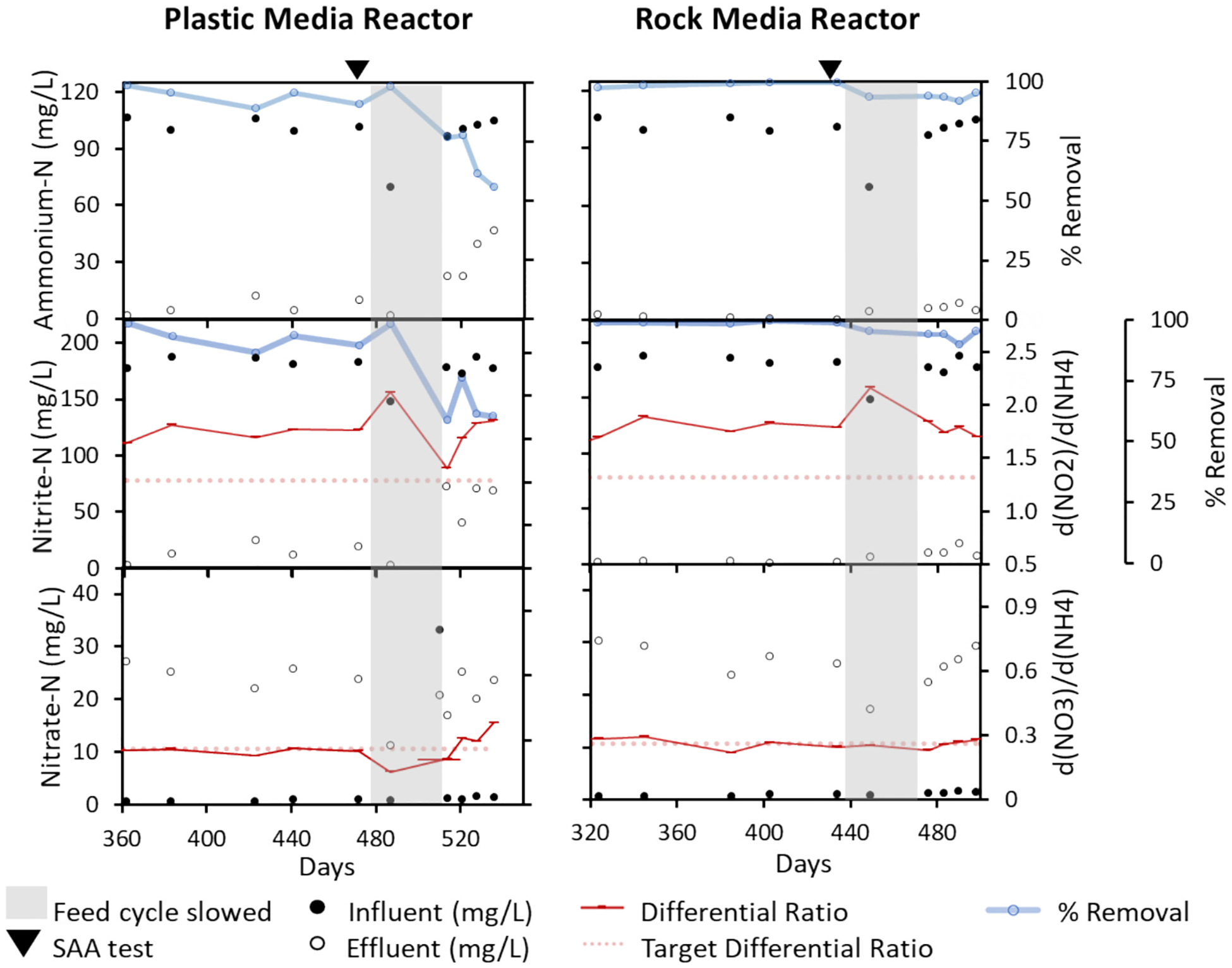
Performance of PMR and RMR after an additional 174 days of operation. A decrease in the frequency of feeding cycles (shaded region) was followed by a return to the regular feeding cycle after day 513 in PMR and day 475 in RMR.

We continued to measure N concentrations from days 361–536 in PMR and day 324–498 in RMR, when performance monitoring was less frequent (Fig. 2). During this longer-term operation, we observed greater N removal in RMR (>90 %) than in PMR. In addition, the switch from daily feeding cycles to only twice/week feeding cycles (from day 475 to 513 in PMR and 437 to 475 in RMR), resulted in reduced nitrogen removal in PMR, which fell to 55 % ${\text{NH}}_{4}^{+}-\text{N}$ removal and 59 % ${\text{NO}}_{2}^{-}-\text{N}$ removal and did not recover to >90 % over the time of this study. By contrast, N removal rates were only slightly reduced in RMR, further reflecting the resilience of biomass formed on the higher surface area lava rock, compared to a more fragile biomass on plastic media (Yang et al., 2021). The iron content of lava rock also may lead to more positively charged sites available for bacterial adhesion (Fofana et al., 2022).

Reactors were opened and biomass was removed from both reactors for the SAA test on the same date, which was day 473 in PMR and day 435 in RMR. The disturbance during biomass removal may also have contributed to the drop in performance of PMR. Nevertheless, the SAA of the combined biomass showed substantial N removal. SAA was highest after 24 h of incubation, with an average of 0.51 g N/g VSS·d ± 0.28 g N/g VSS·d. SAA dropped to 0.16 g N/g VSS·d after 48 h of incubation and 0.17 g N/g VSS·d after 72 h of incubation (Table S2). The 24 h incubation SAA rates are at the high end of values reported in the literature (e.g., He et al., 2018) and signal a functioning anammox community.

### 3.2. Microbial community composition

**Fig. 3.**
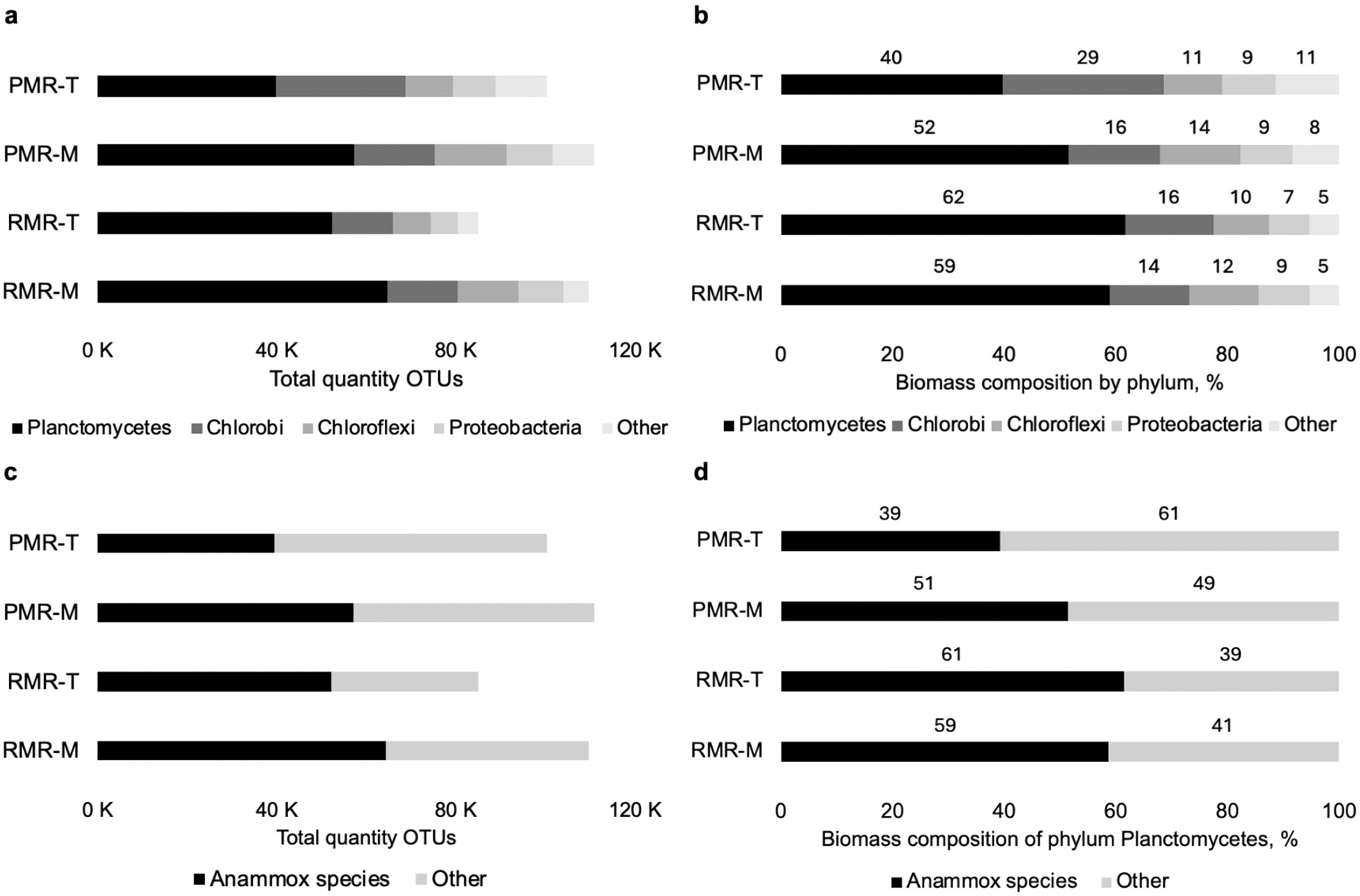
Total quantified operational taxonomic units (OTUs) and relative abundance (%) of the four main phyla (a-b), and within the Planctomycetes phylum (c-d) for samples collected from the top and middle of plastic media reactor (PMR-T and PMR-M, respectively) and the top and middle of the rock media reactor (RMR-T and RMR-M, respectively).

Analysis of biomass from middle and top sections of the reactors following completion of the start-up phase revealed that anammox was successfully enriched in both bioreactors, which shared comparable microbial communities (Fig. 3). The total number of positively identified OTUs were 16 % higher in the middle of reactors than in the top portions, demonstrating higher establishment of bacterial communities with distance from the point of influent. This difference in communities between reactor layers indicates that important nitrogen-removal processes could be responding to gradients within the reactor, with potential depletion of important nutrients or presence of oxygen in the headspace likely impacting communities located near the point of effluent. Further, PMR exhibited 7.8 % higher OTUs than RMR, which likely indicated greater bacterial community establishment during the 38 d difference in start-up time.

Phylum Planctomycete (40–62 %), encompassing anammox bacteria, was predominant in all samples, followed by Chlorobi (14–30 %), Chloroflexi (10–14 %), and Proteobacteria (7–9 %). In both reactors, *Candidatus brocadia sinica* was present (Supplemental Table SI-2.1). It was the only anammox species in the RMR, and its abundance was ~10 % lower in the PMR than in the RMR. PMR additionally contained a very low amount of *Candidatus brocadia fulgida* (0.02 % in the top and middle reactor samples). The overall higher amounts of *C. brocadia* in RMR than PMR may support previously reported findings that high surface area carriers (sponge) were more effective than plastic carrier material at supporting anammox processes (Yang et al., 2021). The synergistic relationships between anammox bacteria and microbes of Chlorobi, Chloroflexi, and Proteobacteria phyla, which typically constitute 30–70 % of anammox consortia (Supplemental Table SI-2.2), allow for microbial cross-feedings, especially with respect to carbon and energy sources (Zhao et al., 2018). While filamentous microbes of the Chlorobi and Chloroflexi phyla are not directly involved in the anammox process, it is hypothesized that they contribute to the structural integrity of the anammox aggregates (Park et al., 2010). Chlorobi microorganisms, in particular, can decompose the extracellular proteins and peptides released by the anammox bacteria, which are then utilized by either the Chlorobi or by other community microbes that are incapable of synthesizing amino acids (Lawson et al., 2017). Not least importantly, Proteobacteria microorganisms, which were present at similar amounts in both reactors (Fig. 3), have been shown to be integral to sustainment of anammox reactors, as they possess genes that anammox bacteria lack for synthesis of certain co-factors required for CO_2_ fixation by anammox (Lawson et al., 2017). As a result, these secondary metabolites from Proteobacteria microorganisms are vital for anammox growth. Several archaea were also identified, including the ammonia-oxidizing *Candidatus nitrosoarchaeum limnia,* and the hydrogenotrophic methanogen *Methanolinea mesophila* (Supplemental Table SI-2.3).

### 3.3. Monitoring parameters that track anammox growth

**Fig. 4.**
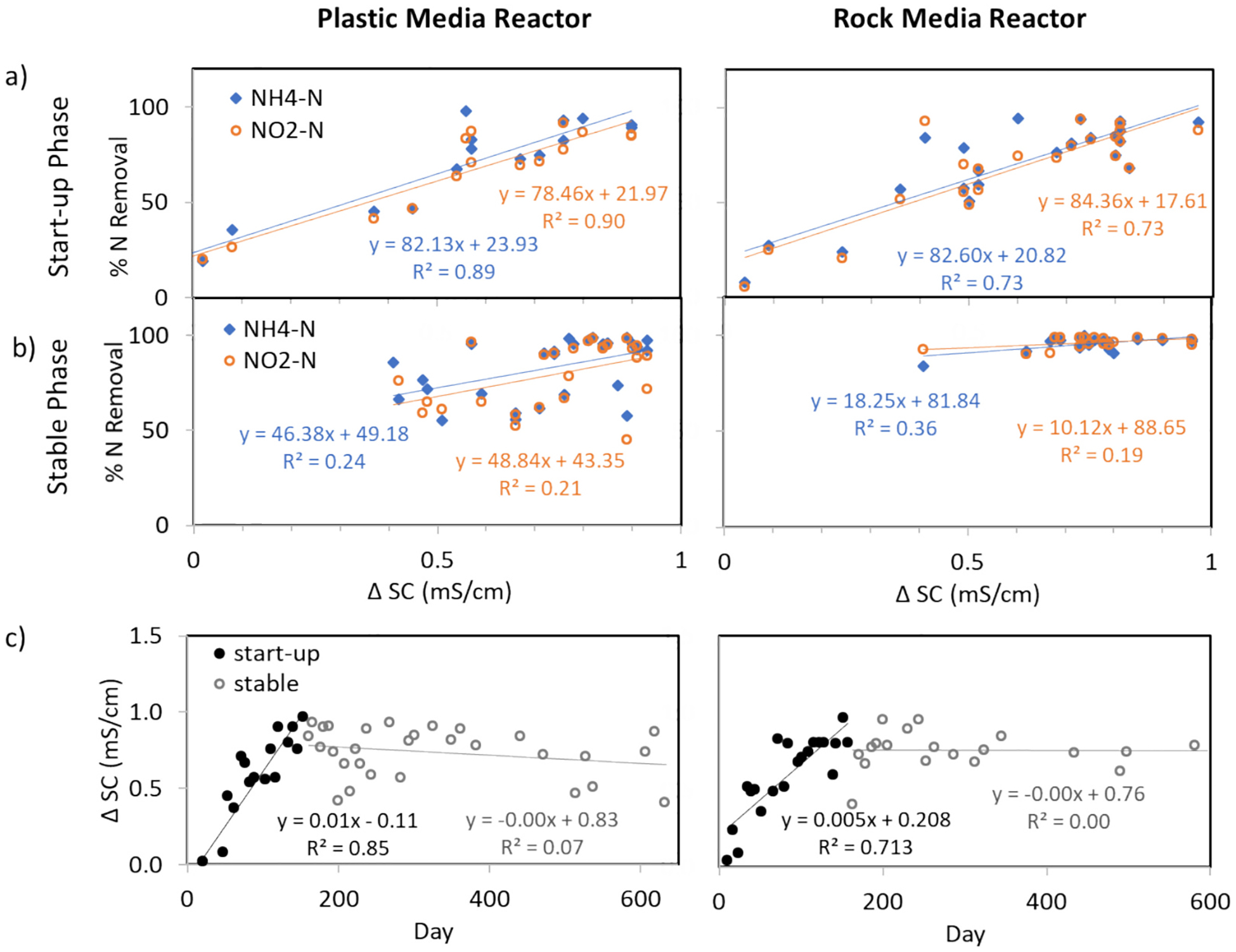
Removal (%) of nutrients plotted vs. differential specific conductivity for a) start-up phase and b) stable phase in PMR and RMR. Monitoring of c) the change in specific conductivity over time shows the gradual increase in Δ SC during start-up in both reactors.

In both reactors, the reduction in specific conductivity from influent to effluent had a strong positive correlation with ammonium-N and nitrite-N uptake rates during the start-up phase, but not in the stable phase (Fig. 4). The gradual reduction of specific conductivity during start-up suggests that anions and cations are removed in proportion to the increase in microbial populations within the reactor. However, during the stable period after anammox enrichment (Fig. 4b), this relationship between differential specific conductivity (Δ SC) and nutrient removal was no longer significant, as per the Spearman correlation test results (Table 1). In the stable phase, the Δ SC in both reactors was maintained at ~0.8 mS/cm (or a reduction from ~3.7 μS/cm in both reactor influents to ~2.9 μS/cm in both reactor effluents). With the exception of one inhibition event in PMR on day 202 (Fig. 1) (due to physical disruption during DNA extraction), nutrient removal rates remained consistently above 90 %, suggesting that a stable microbial community size utilizes the anions (e.g., nitrite) and cations (e.g., ammonium) that primarily contribute to specific conductivity at a steady rate. Therefore, we conclude that the Δ SC may be used during reactor start-up as a proxy for anammox activity, as suggested by previous studies (Li et al., 2012; Yu and Jin, 2012). However, during stable operation, the Δ SC should remain constant, reaching a target value that is representative of optimal performance (Fig. 4c). In our study, the target Δ SC value was ~0.76 mS/cm (where SC of reactor effluent was < 2.9 mS/cm). For the RMR, a constant Δ SC value signified >90 % removal during the stable phase, but for PMR, the Δ SC value was very variable, indicating greater susceptibility to inhibition events and other disturbances, discussed earlier. Target Δ SC values will vary depending on the chemistry of the feed solutions, and it should be noted that for higher salinity wastewaters, the reduction of SC may be less noticeable. The applicability of Δ SC as a performance indicator in anammox enrichment feed solutions with different salinities and in real wastewater merits further research.

**Fig. 5.**
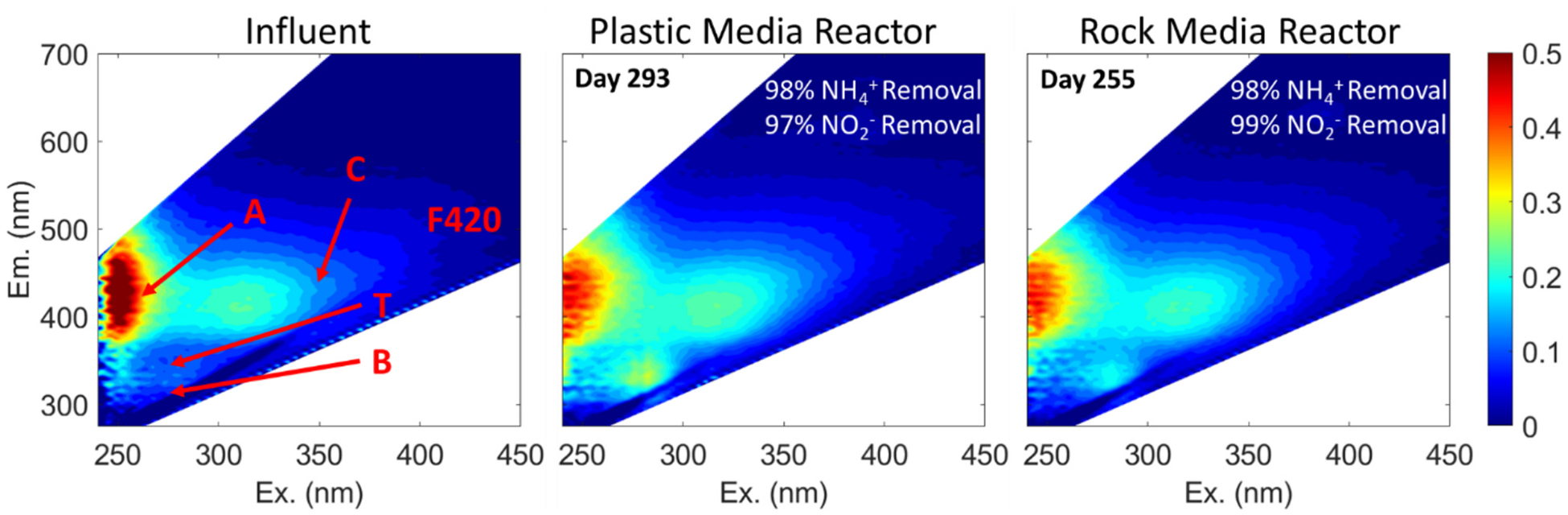
Representative EEMs of influent and PMR and RMR effluent.

There were also significant correlations between fluorescence and nutrient removal in both reactors (Table 1). The feed solution, although low in carbonaceous compounds, did exhibit fluorescence in the A and C region, likely due to the presence of EDTA disodium salt, and was devoid of Peak T and B fluorescence (Fig. 5). The feed solution we used, which is widely used for anammox enrichment and comprises EDTA salt and trace minerals recommended by Strous et al. (1998) and other early studies (Van de Graaf et al., 1995, Dapena-Mora et al., 2007) was found to be fluorescent (Supplemental Fig. S3). Therefore, the changes in fluorescence indices from influent to effluent, which we found to be most responsive to anammox enrichment, are relevant to other enrichment studies using nutrient feed solutions containing EDTA. During reactor start-up, Peaks T and B, which reflect amino acid-like fluorescence, presumably from the microbial biomass, increased in both reactors (Fig. 5), resulting in increases in the effluent/influent ratio of FrI.

Consistent with our observations, correlation analysis showed that increases in the effluent/influent ratio of Freshness Index (FrI) and reduction of Peak A were the most correlated with nutrient uptake for both reactors. During startup, there was a positive correlation between the ratio of FrI of effluent/influent and ${\text{NH}}_{4}^{+}$ removal (R^2^ = 0.75, *p* < 0.001 for PMR, and R^2^ = 0.83, p < 0.001 for RMR; Table 1 shows R values only). The ratio of Peak A of effluent/influent also was significantly related to ${\text{NH}}_{4}^{+}$ removal (R^2^ = 0.72; p < 0.001 for PMR and R^2^ = 0.66; p < 0.001 for RMR; Table 1 shows R values only) but the relationship was negative. The increases in FrI in the effluent of both reactors likely reflect the growing microbial community and its increased production of amino acids and other biological byproducts, which result in a higher FrI (Parlanti et al., 2000). Also, the decrease in Peak A in the effluent of both reactors over time suggests that the more humified inoculum (sludge) within reactors was replaced with autotrophic microbes during the start-up period when reactors became anammox dominant (Hansen et al., 2016).

As in the anammox enrichment study of Ruscalleda et al. (2014), a fluorescence shoulder (denoted as “F420”) previously linked to Coenzyme F420 produced during methanogenesis was also observed at Ex/Em = 420/460–490 nm (Fig. 5), although abundance of methanogenic archaea was <0.02 % in both reactors (Supplemental Table SI-2.3). The increase in this peak was significantly correlated with both ${\text{NH}}_{4}^{+}-\text{N}$ and ${\text{NO}}_{2}^{-}-\text{N}$ removal, but only for PMR, which contained plastic media.

**Fig. 6.**
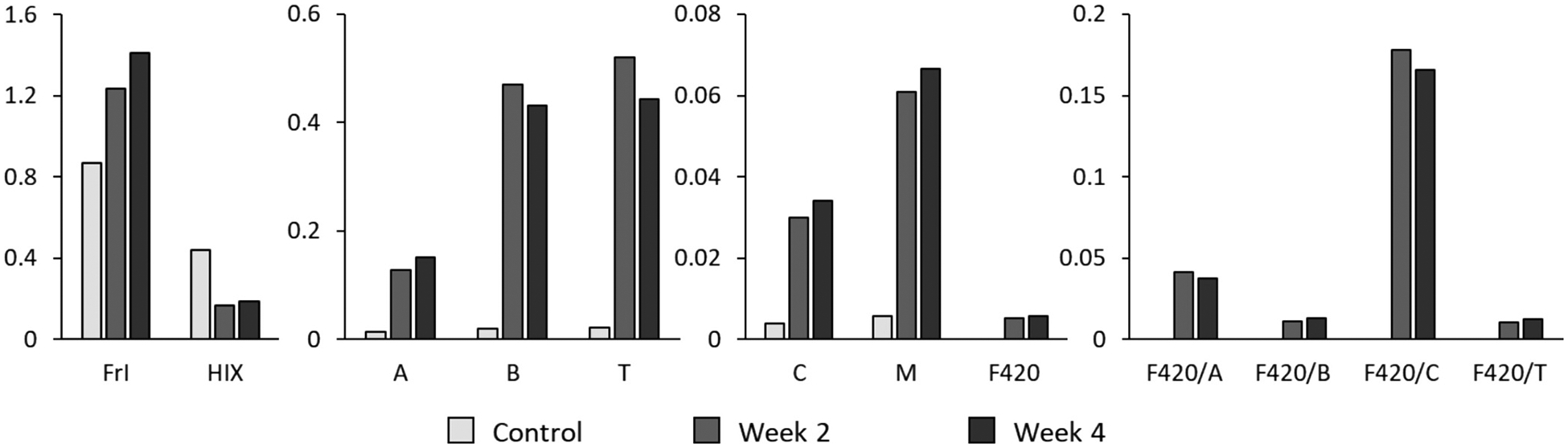
Changes in fluorescence indices (FrI and HIX), peak intensities (Peaks A, B, T, C, M, and F420) and ratios (F420/A, F420/B, F420/C, and F420/T) of plastic media leachates in ultrapure water over time.

In addition, positive correlations were observed between intensities of Peaks B, T, and C and both nutrient removal and time during the start-up phase of PMR, but not RMR, with the strongest correlations seen with time of enrichment. Therefore, an additional experiment was conducted to test for leaching of compounds from plastic media used in PMR (described in Supplemental Methods 1). The experiment confirmed an increase in Peak F420, but also Peaks B, T, and C intensities over time (Fig. 6; Supplemental Table S3), suggesting that carrier media in PMR likely contributed to the more significant correlation between Peak F420 and nutrient removal and to fluorescence differences observed between the PMR and RMR.

## 4. Conclusions

In the current study, anammox enrichment was conducted in up-flow reactors with biomass carriers under low maintenance conditions and using a simple set-up not requiring a sophisticated control unit. Anammox enrichment (sustained at >90 % nutrient removal) was accomplished more rapidly in PMR than in RMR (at 147 d in PMR compared to 171 d in RMR). Nevertheless, both carriers had similar microbial community composition and were resilient to temporary oxygen inhibition during the stable phase, suggesting that accidental oxygen introduction is less impactful for already established anammox consortia. However, after longer term operation (>300 days), the reactor containing plastic media as a carrier was unable to recover from disturbance and altered feeding cycles. By contrast, the lava rock reactor maintained >90 % removal over this period, suggesting that volcanic rock may be a more sustainable carrier for anammox biomass in closed reactor systems.

We also found fluorescence indices (freshness index and Peak A) and Δ SC to be robust and useful indicators for tracking reactor performance during anammox start-up. However, during the stable phase, monitoring increases or decreases in these metrics was less effective for tracking N removal or decreased performance due to inhibition. Instead, target Δ SC values, indicative of optimal performance, should be tracked during stable operation. Fluorescence and specific conductivity do not require extensive sample preparation or chemical reagents, and both can be monitored with lower cost, portable instruments, which may bring anammox enrichment within greater reach in both research and implementation in wastewater treatment systems.

## Supplementary Material

Supplementary File 1

Supplementary File 2

## Figures and Tables

**Table 1:** Spearman’s bivariate correlation analysis showing relationships^1^ (R values) and their significance^2^ between nutrient removal or time of enrichment and specific conductivity (SC) removal, ratios of effluent/influent FrI and HIX, and fluorescent peak intensities.

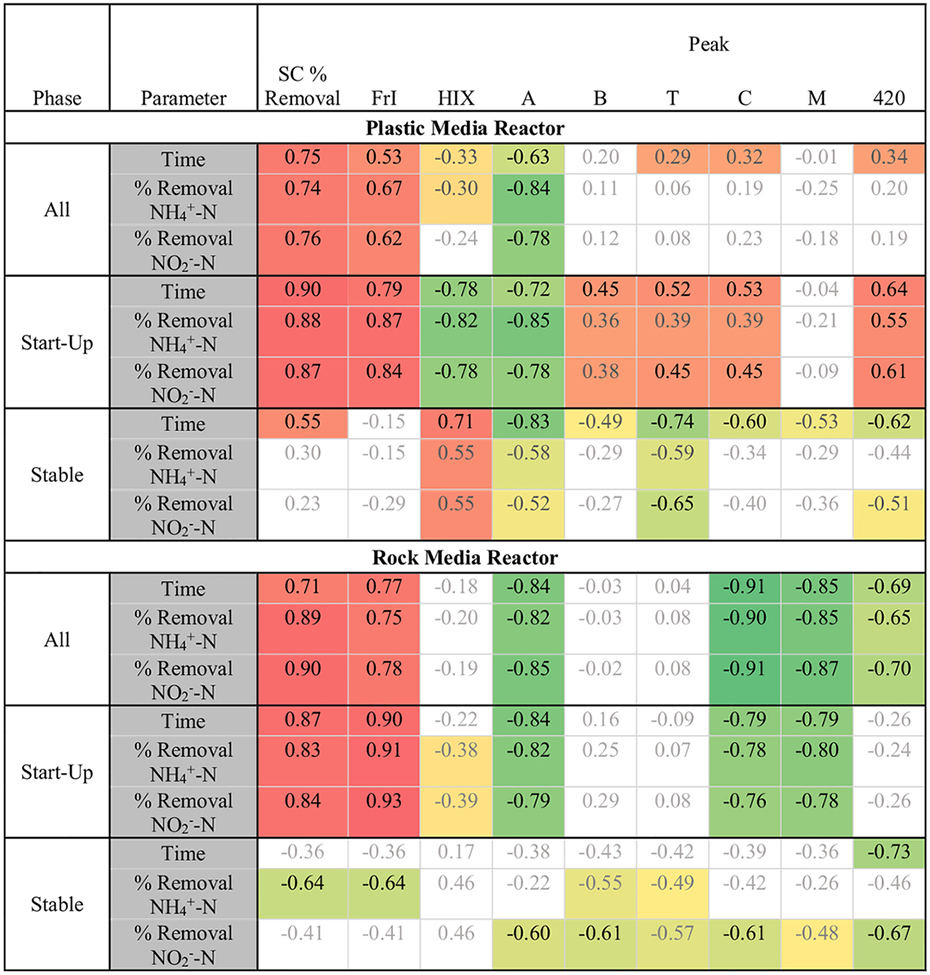

1 Color scale indicates positive (red) to negative (green) correlations.

2 Bold, black: *p* < 0.01; bold, grey: *p* < 0.05; non-bold, grey: no significance.

## Data Availability

Data will be made available on request.

## Appendix A. Supplementary data

Supplementary data to this article can be found online at https://doi.org/10.1016/j.biteb.2025.102081.
