# Supplementary material for: Enrichment of anammox bacteria in up-flow bioreactors enhanced with plastic and rock media: Long-term performance monitoring with fluorescence and specific conductivity
Source: Bioresour Technol Rep. Author manuscript; Available in PMC 2025 Nov 27. (PMC12652372; doi:10.1016/j.biteb.2025.102081)
Supplement: Supplementary File 1 [file NIHMS2116642-supplement-Supplementary_File_1.pdf]

Supplemental Information – 1

Enrichment of anammox bacteria in up-flow bioreactors enhanced with plastic and rock media: long-term performance monitoring with fluorescence and specific conductivity

Polina Popova[1,2], Lilith Astete Vasquez[1], Shiloh Bolden[1], Natalie Mladenov[1]*

[1] Department of Civil, Construction, and Environmental Engineering, San Diego State University

5500 Campanile Dr., San Diego, CA 92182, USA

[2] Now at: Department of Chemical and Environmental Engineering, Yale University, New Haven, CT 06520, USA

*Corresponding author email: nmladenov@sdsu.edu

Supplemental Methods

1. Plastic Leaching Experiment

   To evaluate the contributions of polyester polymer to the fluorescence of the plastic media reactor (PMR), a leaching experiment was conducted. One 1L glass beaker of ultrapure water contained pristine cylindrical polyester plastic hair rollers (Conair brand) used in the setup of the PMR reactor. A second beaker contained only ultrapure water and served as the control. Both beakers were sealed with parafilm and left exposed to the ambient light of the laboratory environment. Fluorescence analysis was conducted at 2 and 4 weeks, as detailed in Methods 2.3.

Supplemental Tables

Table S1. Chemical composition of synthetic wastewater and trace elements solution.

Compound	Concentration
Nutrients in synthetic wastewater	
$(NH_4)_2SO_4$	0.47 g/L
$NaNO_2$	0.59 g/L
$Na_2EDTA*2H_2O$	0.007961 g/L
$KHCO_3$	1.25 g/L
NaH_2PO_4	0.05 g/L
$CaCl_2*2H_2O$	0.0237 g/L
$MgSO_4*7H_2O$	0.2 g/L
$FeSO_4*7H_2O$	0.011 g/L
Trace element solution	1.25 mL/L
Trace element solution composition	
$Na_2EDTA*2H_2O$	19.11 g/L
$ZnCl_2$	0.24 g/L
$CoCl_2*6H_2O$	0.24 g/L
$MnSO_4*H_2O$	0.85 g/L
$CuSO_4*5H_2O$	0.25 g/L
$Na_2MoO_4*2H_2O$	0.21 g/L
$NiCl_2$	0.10 g/L
H_3BO_3	0.01 g/L

Table S2. Specific anammox activity of combined biomass from PMR and RMR, conducted on day 475.

Incubation time (d)	Total N removed (mg/L·d)		SAA (mg N/mg VSS·d)		SAA (mg N/g VSS·h)	
	mean	stdev	mean	stdev	mean	stdev
1	47.9	26.5	0.51	0.28	21.3	11.6
2	15.7	4.7	0.16	0.03	6.67	1.25
3	25.6	9.9	0.17	0.06	7.08	2.50

Table S3. Changes in fluorescence indices (FrI and HIX), peak intensities (Peaks A, B, T, C, M, and F420) and ratios (F420/A, F420/B, F420/C, and F420/T) of leachates of plastic media (hair rollers) soaked in ultrapure water over time.

	Sample		
Parameter	Control	Soaked for 2 weeks	Soaked for 4 weeks
FrI	0.866505	1.235304	1.413445
HIX	0.441947	0.166725	0.190224
A	0.01464	0.128923	0.150782
B	0.020353	0.470553	0.431214
T	0.021006	0.520379	0.443521
C	0.003988	0.030092	0.034217
M	0.005742	0.060883	0.066674
F420	0	0.005353	0.00569
F420/A	0	0.041521	0.037737
F420/B	0	0.011376	0.013195
F420/C	0	0.010287	0.012829
F420/T	0	0.177888	0.166292

Table S4. Spearman's Bivariate Correlation Analysis: Relationship Between Effluent Fluorescence Indices and Nutrient Removal and Time.

Phase	Parameter	Peak/Peak (effluent/effluent)			
		F420/A	F420/B	F420/C	F420/T
Plastic Media Reactor					
All	Time	0.42	-0.44	-0.11	-0.45
	% Removal NH_4^+-N	0.41	-0.07	-0.28	-0.06
	% Removal NO_2^--N	0.36	-0.15	-0.31	-0.14
Start-Up	Time	0.89	0.41	0.33	0.41
	% Removal NH_4^+-N	0.83	0.43	0.18	0.47
	% Removal NO_2^--N	0.84	0.35	0.20	0.40
Stable	Time	-0.46	0.11	-0.52	0.48
	% Removal NH_4^+-N	-0.46	0.18	-0.55	0.36
	% Removal NO_2^--N	-0.54	0.22	-0.63	0.40
Rock Media Reactor					
All	Time	-0.58	-0.64	0.24	-0.70
	% Removal NH_4^+-N	-0.58	-0.65	0.25	-0.70
	% Removal NO_2^--N	-0.58	-0.64	0.25	-0.69
Start-Up	Time	0.16	-0.01	0.61	-0.24
	% Removal NH_4^+-N	0.12	-0.11	0.61	-0.32
	% Removal NO_2^--N	0.14	-0.06	0.63	-0.27
Stable	Time	-0.62	-0.40	-0.59	-0.35
	% Removal NH_4^+-N	-0.47	-0.25	-0.48	-0.16
	% Removal NO_2^--N	-0.52	-0.27	-0.48	-0.14

Bold, black: $p < 0.01$
Bold, grey: $p < 0.05$
Non-bold, grey: no significance

Supplemental Figures

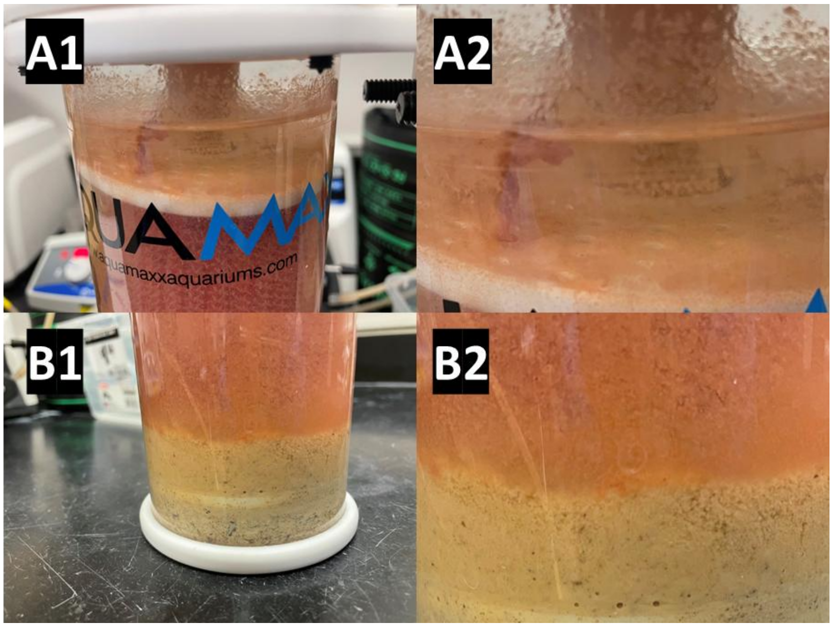

Figure S1. Visual evidence of anammox within PMR on day 148: A1 – floating anammox floc and biofilm on reactor wall in the top section of reactor, A2 – magnified view of A1, B1 – anammox biofilm on reactor wall in the body of reactor, B2 – magnified view of B1.

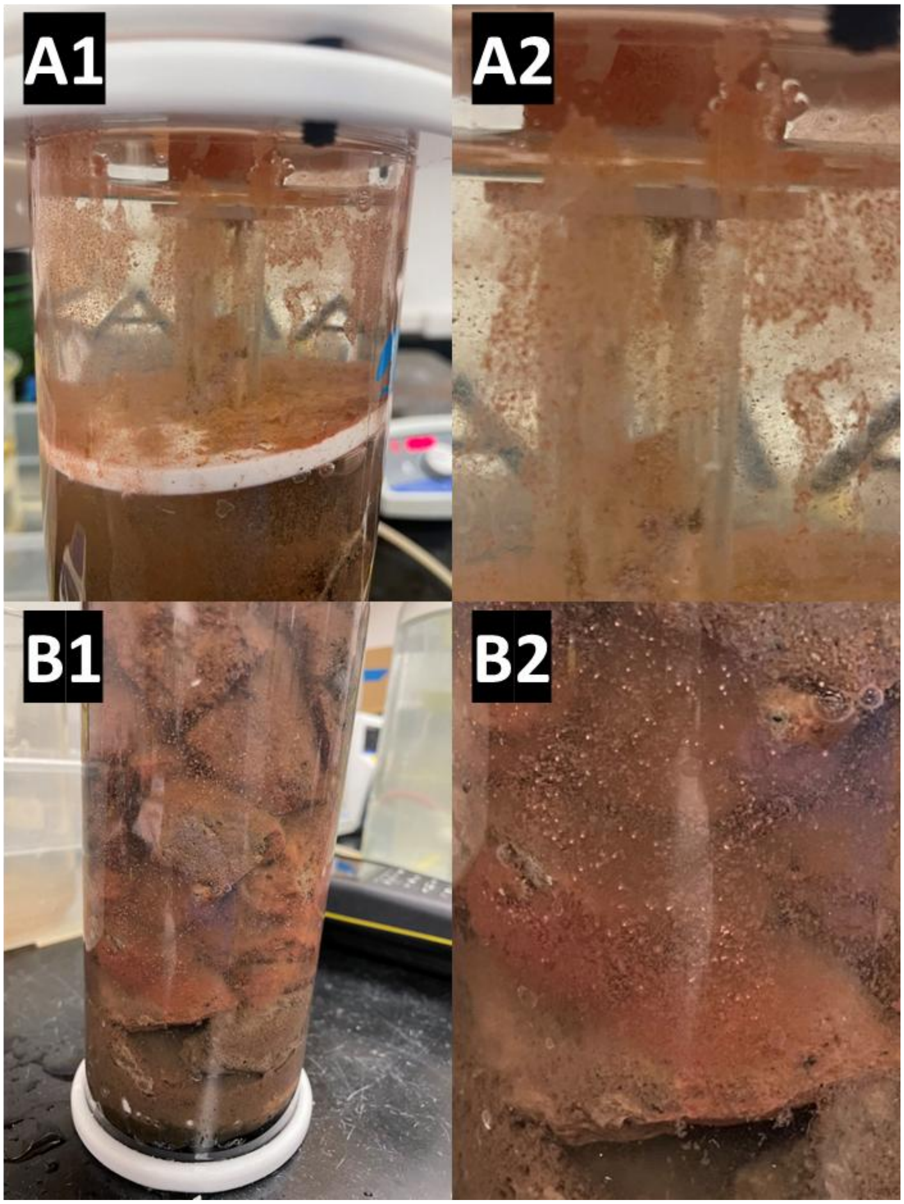

Figure S2. Visual evidence of anammox within RMR on days 170 (A1, A2) and 175 (B1 and B2): A1 – floating anammox floc and biofilm on reactor wall in the top section of reactor, A2 – magnified view of A1, B1 – anammox biofilm on reactor wall and porous media in the body of reactor, B2 – magnified view of B1.

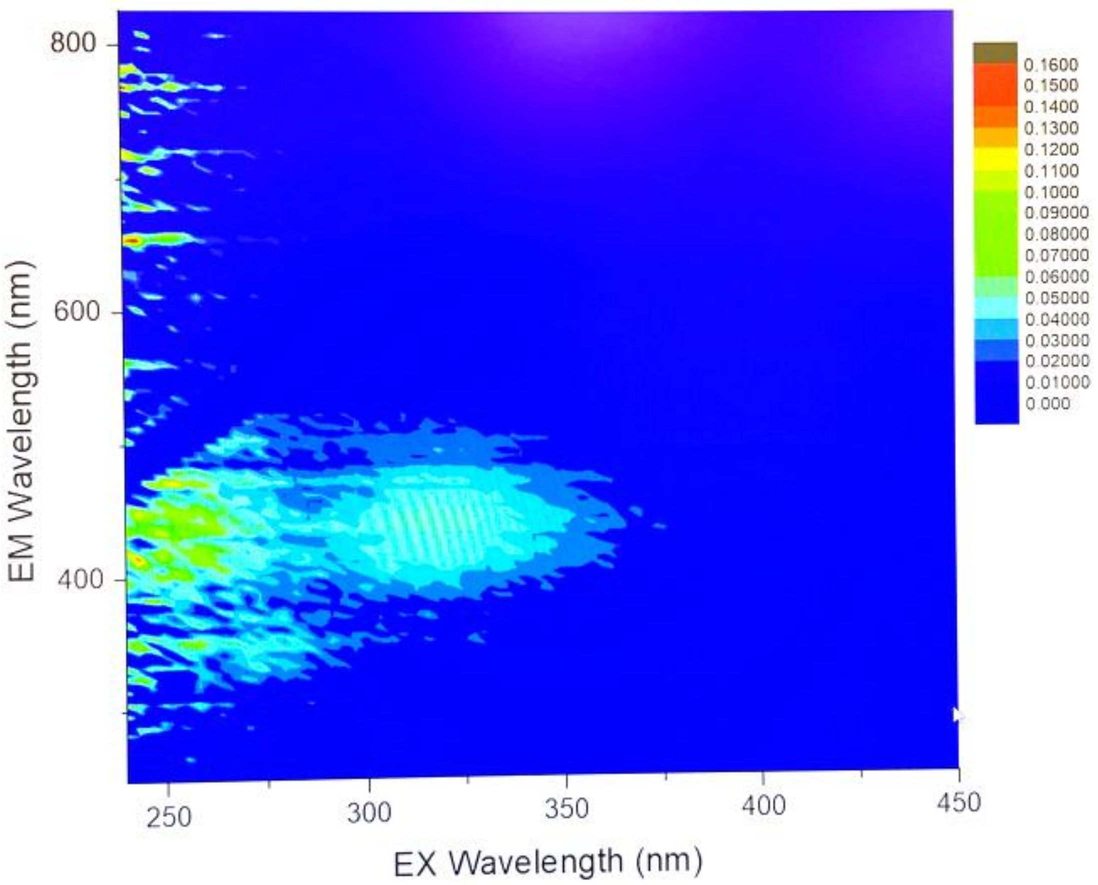

Figure S3. Three-dimensional excitation emission matrix spectrum of EDTA disodium salt ($Na_2EDTA*2H_2O$) at a concentration of 8.0 mg/L in ultrapure water. Colorbar shows corrected fluorescence intensities in Raman Units (RU).
