# Supplementary material for: Enrichment of anammox bacteria in up-flow bioreactors enhanced with plastic and rock media: Long-term performance monitoring with fluorescence and specific conductivity
Source: Bioresour Technol Rep. Author manuscript; Available in PMC 2025 Nov 27. (PMC12652372; doi:10.1016/j.biteb.2025.102081)
Supplement: Supplementary File 2 [file NIHMS2116642-supplement-Supplementary_File_2.pdf]

Supplemental Information – 2

Enrichment of anammox bacteria in up-flow bioreactors enhanced with plastic and rock media: long-term performance monitoring with fluorescence and specific conductivity

Polina Popova[1,2], Lilith Astete Vasquez[1], Shiloh Bolden[1], Natalie Mladenov[1]*

[1] Department of Civil, Construction, and Environmental Engineering, San Diego State University
5500 Campanile Dr., San Diego, CA 92182, USA
[2] Now at: Department of Chemical and Environmental Engineering, Yale University, New Haven, CT 06520, USA

*Corresponding author email: nmladenov@sdsu.edu

Table of Contents

Table SI-2.1. Bacteria species in anammox reactors

Description: Operational taxonomic units (OTU) and percent of total consortia (%) for bacteria from anaerobic ammonium oxidation (anammox) enrichment reactors at the species level. Biomass extracted from plastic media (PMR) and rock media (RMR) reactors at top (T) and middle (M) layers. Species with 0% were omitted from list of taxonomic results.

Table SI-2.2. Bacteria phyla in anammox reactors

Description: OTU and percent of total consortia (%) for bacteria from anammox enrichment reactors at the phylum level. Biomass extracted from plastic media (PMR) and rock media (RMR) reactors at top (T) and middle (M) layers. Phyla with 0% were omitted from list of taxonomic results.

Table SI-2.3. Archaea species in anammox reactors

Description: OTU and percent of total consortia (%) for archaea from anaerobic ammonium oxidation (anammox) enrichment reactors at the species level. Biomass extracted from plastic media (PMR) and rock media (RMR) reactors at top (T) and middle (M) layers. Species with 0% were omitted from list of taxonomic results.

Table SI-2.1. Bacteria species in anammox reactors

Species	PMR-T		PMR-M		RMR-T		RMR-M	
	OTU	%	OTU	%	OTU	%	OTU	%
Candidatus brocadia sinica	39350	39.2	57003	51.4	52181	61.5	64363	58.7
Prosthecochloris indica	28944	28.9	18120	16.4	13461	15.9	15637	14.3
Thermanaerothrix daxensis	6905	6.9	8791	7.9	2863	3.4	5699	5.2
Zoogloea ramigera	4612	4.6	6042	5.5	2529	3.0	3438	3.1
Ornatilinea apprima	2284	2.3	988	0.9	4284	5.0	6048	5.5
Fimbriimonas ginsengisoli	1483	1.5	4336	3.9	2336	2.8	2455	2.2
Heliobacterium chlorum	2248	2.2	1786	1.6	991	1.2	1408	1.3
Longilinea arvoryzae	262	0.3	2681	2.4	1019	1.2	1337	1.2
Ignavibacterium album	3140	3.1	790	0.7	318	0.4	878	0.8
Bellilinea caldifistulae	835	0.8	3344	3.0	225	0.3	350	0.3
Sulfurisoma sediminicola	626	0.6	783	0.7	977	1.2	1920	1.8
Sulfuritalea hydrogenivorans	550	0.5	1129	1.0	695	0.8	1848	1.7
Calditerricola yamamurae	1642	1.6	962	0.9	151	0.2	147	0.1
Arhodomonas recens	162	0.2	229	0.2	496	0.6	390	0.4
Candidatus solibacter usitatus	594	0.6	326	0.3	106	0.1	192	0.2
Thauera mechernichensis	564	0.6	99	0.1	147	0.2	140	0.1
Rhodoplanes elegans	449	0.4	241	0.2	85	0.1	131	0.1
Candidatus nitrotoga arctica	150	0.1	198	0.2	155	0.2	279	0.3
Aetherobacter rufus	489	0.5	89	0.1	111	0.1	90	0.1
Gemmatimonas aurantiaca	583	0.6	82	0.1	6	0.0	33	0.0
Skermanella xinjiangensis	261	0.3	275	0.2	59	0.1	61	0.1
Candidatus planktothricoides rosea	217	0.2	193	0.2	109	0.1	103	0.1
Aridibacter acidobacteria bacterium	514	0.5	25	0.0	4	0.0	15	0.0
Herminiimonas saxobsidens	211	0.2	22	0.0	167	0.2	135	0.1
Aciditerrimonas ferrireducens	74	0.1	248	0.2	93	0.1	58	0.1
Edaphobacter modestum	122	0.1	88	0.1	82	0.1	116	0.1
Planctomyces brasiliensis	65	0.1	40	0.0	117	0.1	153	0.1
Polyangium spumosum	0	0.0	4	0.0	46	0.1	311	0.3
Thioprofundum hispidum	172	0.2	127	0.1	11	0.0	42	0.0
Chromobacterium suttsuga	39	0.0	66	0.1	118	0.1	97	0.1
Litorilinea aerophila	93	0.1	79	0.1	34	0.0	83	0.1
Gaiella occulta	118	0.1	79	0.1	41	0.0	24	0.0
Georgfuchsia toluolica	16	0.0	10	0.0	26	0.0	176	0.2
Bythopirellula goksoyri	197	0.2	15	0.0	1	0.0	4	0.0
Sphaerobacter thermophilus	135	0.1	12	0.0	6	0.0	22	0.0
Elioraea tepidiphila	37	0.0	84	0.1	24	0.0	26	0.0
Candidatus brocadia brasiliensis	63	0.1	20	0.0	31	0.0	49	0.0
Hyphomicrobium aestuarii	61	0.1	55	0.0	16	0.0	25	0.0
Thiohalomonas denitrificans	84	0.1	0	0.0	42	0.0	30	0.0
Caldilinea aerophila	30	0.0	52	0.0	14	0.0	57	0.1
Melioribacter roseus	19	0.0	14	0.0	71	0.1	42	0.0
Pedomicrobium australicum	89	0.1	10	0.0	20	0.0	23	0.0
Limnobacter litoralis	76	0.1	53	0.0	2	0.0	8	0.0
Desulfonatronum thiosulfatophilum	28	0.0	31	0.0	10	0.0	69	0.1
Pseudolabrys taiwanensis	18	0.0	27	0.0	46	0.1	35	0.0
Gulbenkiania mobilis	35	0.0	23	0.0	10	0.0	55	0.1

Truepera radiovictrix	84	0.1	24	0.0	6	0.0	4	0.0
Candidatus nitrospira defluvii	94	0.1	11	0.0	4	0.0	4	0.0
Pseudohongiella gamma proteobacterium	55	0.1	18	0.0	21	0.0	17	0.0
Rhodocyclus tenuis	12	0.0	3	0.0	45	0.1	48	0.0
Vogesella indigofera	42	0.0	13	0.0	20	0.0	32	0.0
Lysobacter arseniciresistens	1	0.0	100	0.1	1	0.0	2	0.0
Pedosphaera parvula	53	0.1	17	0.0	16	0.0	18	0.0
Candidatus brocadia caroliniensis	18	0.0	37	0.0	10	0.0	28	0.0
Caldibacillus geobacillus debilis	40	0.0	17	0.0	12	0.0	17	0.0
Thiobacillus thioparus	0	0.0	3	0.0	5	0.0	76	0.1
Bradyrhizobium lupini	5	0.0	43	0.0	12	0.0	23	0.0
Paracoccus aminophilus	18	0.0	17	0.0	13	0.0	35	0.0
Dokdonella ginsengisoli	10	0.0	15	0.0	17	0.0	38	0.0
Opitutus terrae	53	0.1	7	0.0	10	0.0	6	0.0
Nitrosomonas europaea	39	0.0	12	0.0	2	0.0	23	0.0
Chitinophaga arvensicola	33	0.0	34	0.0	2	0.0	5	0.0
Haliangium ochraceum	66	0.1	5	0.0	0	0.0	2	0.0
Burkholderia multivorans	5	0.0	44	0.0	3	0.0	21	0.0
Hyphomicrobium vulgare	8	0.0	28	0.0	9	0.0	27	0.0
Legionella santicrucis	67	0.1	0	0.0	0	0.0	1	0.0
Bradyrhizobium elkanii	64	0.1	2	0.0	0	0.0	0	0.0
Algisphaera agarilytica	60	0.1	1	0.0	1	0.0	4	0.0
Rhodovibrio salinarum	3	0.0	36	0.0	14	0.0	11	0.0
Nitrosovibrio tenuis	36	0.0	1	0.0	14	0.0	10	0.0
Carboxydocella manganica	14	0.0	14	0.0	25	0.0	7	0.0
Chthoniobacter flavus	52	0.1	1	0.0	4	0.0	1	0.0
Lautropia mirabilis	20	0.0	20	0.0	5	0.0	13	0.0
Sorangium cellulosum	0	0.0	0	0.0	6	0.0	52	0.0
Mesorhizobium amorphae	3	0.0	28	0.0	11	0.0	10	0.0
Desulfomicrobium apsheronum	37	0.0	8	0.0	2	0.0	4	0.0
Phyllobacterium trifolii	4	0.0	29	0.0	11	0.0	6	0.0
Geofilum rikenellaceae bacterium	24	0.0	0	0.0	4	0.0	20	0.0
Acidibacter gamma proteobacterium	33	0.0	1	0.0	4	0.0	9	0.0
Paracoccus thiocyanatus	22	0.0	3	0.0	16	0.0	5	0.0
Candidatus atelocyanobacterium cyanobacterium ucyn_a	8	0.0	19	0.0	10	0.0	9	0.0
Caldimonas taiwanensis	1	0.0	10	0.0	17	0.0	18	0.0
Mycobacterium smegmatis	28	0.0	11	0.0	1	0.0	5	0.0
Iamia majanohamensis	12	0.0	15	0.0	8	0.0	9	0.0
Acidovorax caeni	3	0.0	20	0.0	2	0.0	17	0.0
Owenweeksia hongkongensis	0	0.0	6	0.0	20	0.0	14	0.0
Desulfatitalea tepidiphila	10	0.0	9	0.0	7	0.0	14	0.0
Pelagicoccus mobilis	25	0.0	14	0.0	0	0.0	0	0.0
Ohtaekwangia koreensis	29	0.0	8	0.0	0	0.0	0	0.0
Mycobacterium ratisbonense	5	0.0	13	0.0	8	0.0	10	0.0
Ureibacillus suwonensis	20	0.0	6	0.0	5	0.0	4	0.0
Hyphomicrobium zavarzinii	8	0.0	18	0.0	5	0.0	4	0.0
Chelatococcus daeguensis	10	0.0	12	0.0	7	0.0	6	0.0
Oligotropha carboxidovorans	6	0.0	13	0.0	7	0.0	9	0.0
Levilinea saccharolytica	13	0.0	2	0.0	8	0.0	12	0.0

Turicibacter sanguinis	0	0.0	10	0.0	4	0.0	20	0.0
Parvibaculum lavamentivorans	12	0.0	18	0.0	2	0.0	1	0.0
Thioalkalivibrio thiocyanodenitrificans	17	0.0	8	0.0	3	0.0	5	0.0
Singulisphaera mucilaginosa	24	0.0	7	0.0	0	0.0	1	0.0
Tepidibacillus fermentans	15	0.0	7	0.0	2	0.0	8	0.0
Alicyclobacillus tolerans	1	0.0	5	0.0	12	0.0	14	0.0
Blastocatella fastidiosa	29	0.0	1	0.0	1	0.0	0	0.0
Anaeromyxobacter dehalogenans	18	0.0	1	0.0	2	0.0	10	0.0
Desulfuromonas palmitatis	10	0.0	7	0.0	4	0.0	10	0.0
Nordella oligomobilis	18	0.0	4	0.0	6	0.0	2	0.0
Nevskia soli	22	0.0	3	0.0	3	0.0	2	0.0
Aquabacterium limnoticum	2	0.0	10	0.0	1	0.0	17	0.0
Pannonibacter phragmitetus	11	0.0	12	0.0	3	0.0	3	0.0
Thauera chlorobenzoica	3	0.0	12	0.0	4	0.0	10	0.0
Pyrinomonas acidobacteriaceae bacterium	28	0.0	0	0.0	0	0.0	0	0.0
Gemmata obscuriglobus	12	0.0	6	0.0	4	0.0	6	0.0
Candidatus desulforudis audaxviator	12	0.0	1	0.0	1	0.0	12	0.0
Clostridium ruminantium	0	0.0	5	0.0	6	0.0	15	0.0
Labrenzia aggregata	13	0.0	5	0.0	3	0.0	3	0.0
Candidatus brocadia fulgida	1	0.0	22	0.0	0	0.0	0	0.0
Thioalkalivibrio paradoxus	9	0.0	7	0.0	4	0.0	3	0.0
Trigonala elaeagnus	6	0.0	8	0.0	3	0.0	6	0.0
Jahnella thaxteri	7	0.0	15	0.0	0	0.0	0	0.0
Conexibacter woesei	14	0.0	5	0.0	0	0.0	3	0.0
Azoarcus indigens	3	0.0	12	0.0	0	0.0	7	0.0
Thermaerobacter nagasakiensis	0	0.0	21	0.0	0	0.0	0	0.0
Sphingopyxis macrogoltabida	1	0.0	14	0.0	1	0.0	4	0.0
Thauera phenylacetica	3	0.0	4	0.0	5	0.0	8	0.0
Rickettsia honei	0	0.0	6	0.0	4	0.0	9	0.0
Bradyrhizobium jicamae	10	0.0	2	0.0	1	0.0	5	0.0
Dolichospermum planctonicum	1	0.0	13	0.0	0	0.0	4	0.0
Mycobacterium flavescens	0	0.0	3	0.0	4	0.0	11	0.0
Devosia insulae	12	0.0	3	0.0	0	0.0	2	0.0
Amorphus coralli	1	0.0	4	0.0	7	0.0	5	0.0
Aquamicrobium aestuarii	4	0.0	8	0.0	0	0.0	4	0.0
Mycobacterium kumamotonense	4	0.0	1	0.0	3	0.0	7	0.0
Clostridium disporicum	1	0.0	5	0.0	4	0.0	4	0.0
Aquabacterium parvum	7	0.0	1	0.0	0	0.0	6	0.0
Alkalitalea saponilacus	0	0.0	0	0.0	2	0.0	12	0.0
Clostridium tunisiense	1	0.0	12	0.0	0	0.0	0	0.0
Nitrobacter winogradskyi	2	0.0	5	0.0	3	0.0	3	0.0
Mycobacterium arupense	0	0.0	3	0.0	3	0.0	7	0.0
Ensifer xinjiangense	2	0.0	6	0.0	2	0.0	2	0.0
Clostridium cellulovorans	0	0.0	1	0.0	7	0.0	4	0.0
Rhodobium orientis	4	0.0	4	0.0	0	0.0	4	0.0
Conexibacter arvalis	8	0.0	2	0.0	0	0.0	0	0.0
Brevibacterium picturae	0	0.0	7	0.0	1	0.0	1	0.0
Dokdonella soli	2	0.0	4	0.0	1	0.0	2	0.0
Leucobacter denitrificans	0	0.0	0	0.0	5	0.0	4	0.0

Methylibium petroleiphilum	2	0.0	2	0.0	1	0.0	4	0.0
Trichococcus pasteurii	0	0.0	8	0.0	0	0.0	0	0.0
Parapusillimonas pusillimonas terrae	6	0.0	1	0.0	0	0.0	1	0.0
Peptoclostridium clostridium difficile	0	0.0	0	0.0	2	0.0	6	0.0
Mycobacterium riyadhense	1	0.0	4	0.0	1	0.0	1	0.0
Tetracoccus cechii	0	0.0	6	0.0	0	0.0	1	0.0
Vitreoscilla filiformis	1	0.0	3	0.0	0	0.0	2	0.0
Hyphomicrobium denitrificans	0	0.0	0	0.0	0	0.0	6	0.0
Paracoccus koreensis	3	0.0	0	0.0	0	0.0	2	0.0
Rhodopseudomonas palustris	0	0.0	1	0.0	1	0.0	3	0.0
Clostridium metallolevans	0	0.0	0	0.0	1	0.0	4	0.0
Parachlamydia acanthamoebae	0	0.0	4	0.0	0	0.0	0	0.0
Hyphomicrobium hollandicum	1	0.0	1	0.0	0	0.0	2	0.0
Gordonia bronchialis	0	0.0	0	0.0	1	0.0	3	0.0
Legionella anisa	0	0.0	3	0.0	0	0.0	0	0.0
Aminobacter anthyllidis	0	0.0	3	0.0	0	0.0	0	0.0
Roseomonas stagni	0	0.0	0	0.0	2	0.0	1	0.0
Mitsuaria chitosanitabida	0	0.0	1	0.0	1	0.0	1	0.0
Pelomonas pseudomonas saccharophila	0	0.0	2	0.0	0	0.0	1	0.0
Herbaspirillum massiliense	0	0.0	0	0.0	1	0.0	2	0.0
Nocardioides mesophilus	0	0.0	0	0.0	0	0.0	3	0.0
Shinella zoogloeoides	0	0.0	0	0.0	0	0.0	3	0.0
Nitratireductor lucknowense	2	0.0	0	0.0	0	0.0	0	0.0
Shinella fusca	1	0.0	1	0.0	0	0.0	0	0.0
Micrococcus luteus	0	0.0	2	0.0	0	0.0	0	0.0
Lysinibacillus sphaericus	0	0.0	2	0.0	0	0.0	0	0.0
Peptoclostridium clostridium sordellii	0	0.0	2	0.0	0	0.0	0	0.0
Hydrogenophaga flava	0	0.0	2	0.0	0	0.0	0	0.0
Methylocapsa acidiphila	1	0.0	0	0.0	0	0.0	1	0.0
Comamonas kerstersii	1	0.0	0	0.0	0	0.0	1	0.0
Curvibacter delicatus	1	0.0	0	0.0	0	0.0	1	0.0
Sporichthya brevicatena	0	0.0	1	0.0	0	0.0	1	0.0
Hyphomicrobium facile	0	0.0	1	0.0	0	0.0	1	0.0
Sediminibacterium salmoneum	0	0.0	1	0.0	0	0.0	1	0.0
Limnohabitans curvus	0	0.0	1	0.0	0	0.0	1	0.0
Oxobacter pfennigii	0	0.0	0	0.0	0	0.0	2	0.0
Corynebacterium glutamicum	1	0.0	0	0.0	0	0.0	0	0.0
Rathayibacter tritici	1	0.0	0	0.0	0	0.0	0	0.0
Sinorhizobium ensifer fredii	1	0.0	0	0.0	0	0.0	0	0.0
Sphingobacterium daejeonense	1	0.0	0	0.0	0	0.0	0	0.0
Methylosinus trichosporium	1	0.0	0	0.0	0	0.0	0	0.0
Kocuria rhizophila	1	0.0	0	0.0	0	0.0	0	0.0
Pseudomonas thermaerum	1	0.0	0	0.0	0	0.0	0	0.0
Selenomonas artemidis	0	0.0	1	0.0	0	0.0	0	0.0
Clostridium cadaveris	0	0.0	1	0.0	0	0.0	0	0.0
Corynebacterium tuberculostearicum	0	0.0	1	0.0	0	0.0	0	0.0
Ancylobacter polymorphus	0	0.0	1	0.0	0	0.0	0	0.0
Clostridium thiosulfatireducens	0	0.0	1	0.0	0	0.0	0	0.0
Fulvimonas soli	0	0.0	1	0.0	0	0.0	0	0.0

Bacillus hwajinpoensis	0	0.0	1	0.0	0	0.0	0	0.0
Tissierella praeacuta	0	0.0	1	0.0	0	0.0	0	0.0
Blautia faecis	0	0.0	1	0.0	0	0.0	0	0.0
Rothia mucilaginosa	0	0.0	1	0.0	0	0.0	0	0.0
Heliobacillus mobilis	0	0.0	1	0.0	0	0.0	0	0.0
Bacillus saliphilus	0	0.0	1	0.0	0	0.0	0	0.0
Mycobacterium triplex	0	0.0	0	0.0	0	0.0	1	0.0
Methylobacterium tardum	0	0.0	0	0.0	0	0.0	1	0.0
Clostridium saccharoperbutylacetonicum	0	0.0	0	0.0	0	0.0	1	0.0
Streptomyces glaucescens	0	0.0	0	0.0	0	0.0	1	0.0
Azohydromonas australica	0	0.0	0	0.0	0	0.0	1	0.0
Herbiconiux moechotypicola	0	0.0	0	0.0	0	0.0	1	0.0
Aminobacter aminovorans	0	0.0	0	0.0	0	0.0	1	0.0
Gemmobacter catellibacterium terrae	0	0.0	0	0.0	0	0.0	1	0.0
Bacteroides vulgatus	0	0.0	0	0.0	0	0.0	1	0.0
Rhodovulum kholense	0	0.0	0	0.0	0	0.0	1	0.0
Vasilyevaea enhydra	0	0.0	0	0.0	0	0.0	1	0.0
Streptococcus lutetiensis	0	0.0	0	0.0	0	0.0	1	0.0
Brevibacterium linens	0	0.0	0	0.0	0	0.0	1	0.0
Anaerococcus pacaensis	0	0.0	0	0.0	0	0.0	1	0.0
Pseudogracilibacillus auburnensis	0	0.0	0	0.0	0	0.0	1	0.0
Alsobacter metallidurans	0	0.0	0	0.0	0	0.0	1	0.0
Clostridium paraputrificum	0	0.0	0	0.0	0	0.0	1	0.0
Roseomonas lacus	0	0.0	0	0.0	0	0.0	1	0.0
Roseibacillus ponti	0	0.0	0	0.0	0	0.0	1	0.0
Solirubrobacter ginsenosidimutans	0	0.0	0	0.0	0	0.0	1	0.0

Table SI-2.2. Bacteria phyla in anammox reactors

	PMR-T		PMR-M		RMR-T		RMR-M	
**Phylum**	**OTU**	**%**	**OTU**	**%**	**OTU**	**%**	**OTU**	**%**
Planctomycetes	39790	39.7	57151	51.6	52345	61.7	64608	58.9
Chlorobi	28944	28.9	18120	16.4	13461	15.9	15637	14.3
Chloroflexi	10557	10.5	15949	14.4	8453	10.0	13608	12.4
Proteobacteria	9532	9.5	10313	9.3	6133	7.2	10031	9.1
Armatimonadetes	1483	1.5	4336	3.9	2336	2.8	2455	2.2
Firmicutes	4000	4.0	2880	2.6	1226	1.4	1683	1.5
Ignavibacteriae	3159	3.2	804	0.7	389	0.5	920	0.8
Acidobacteria	1287	1.3	440	0.4	193	0.2	323	0.3
Actinobacteria	267	0.3	396	0.4	169	0.2	152	0.1
Gemmatimonadetes	583	0.6	82	0.1	6	0.0	33	0.0
Cyanobacteria	226	0.2	225	0.2	119	0.1	116	0.1
Verrucomicrobia	183	0.2	39	0.0	30	0.0	26	0.0
Bacteroidetes	87	0.1	49	0.0	28	0.0	53	0.0
Deinococcus_thermus	84	0.1	24	0.0	6	0.0	4	0.0
Nitrospirae	94	0.1	11	0.0	4	0.0	4	0.0
Chlamydiae	0	0.0	4	0.0	0	0.0	0	0.0

Table SI-2.3. Archaea species in anammox reactors

Species	PMR-T		PMR-M		RMR-T		RMR-M	
	**OTU**	**%**	**OTU**	**%**	**OTU**	**%**	**OTU**	**%**
Methanolinea mesophila	0	0.0	2	2.9	17	63.0	85	78.7
Candidatus nitrosoarchaeum limnia	26	100.0	62	89.9	2	7.4	5	4.6
Methanosaeta concilii	0	0.0	0	0.0	8	29.6	18	16.7
Methanosarcina siciliae	0	0.0	5	7.2	0	0.0	0	0.0
